# Round the Hospitals

**Published:** 1916-03-04

**Authors:** 


### ROUND THE HOSPITALS.

The nursing profession is to be congratulated on its good fortune in having attracted the interest and experience of the Hon. Arthur Stanley to pilot it safely out of chaos to order. At a remarkable meeting of the representatives of various societies with which nurses have associated themselves, matrons and nurses representing important centres throughout Great Britain and Ireland, held at 83 Pall Mall on February 23, Mr. Stanley proved to be so well informed, practical, and courteous a chairman that the meeting ended in agreement. This was shown at the end by the fact that a vote of acknowledgment to Mr. Stanley was carried unanimously with cheers. There are to be further meetings with Mr. Stanley on the part of those who claim to be representatives of real nursing opinion, and it is not too much to say that Mr. Stanley is justified in hoping—indeed, we may say in expecting—that the scheme of the College of Nursing is already so generally supported by the unprejudiced nurses of all sections as to constitute practical unanimity. Sir Charles Russell's speech at the meeting, it will be seen, is full of wise counsel to nurses in the present crisis.

One advantage which sisters and charge nurses, or many of them, have gained from the war has been an opportunity, owing to the shortage in the resident medical staff, to make themselves helpful to the honorary medical staff in both medical and surgical wards. In one hospital in the South of England the sisters have learnt how to take the history of patients, to assist in the laboratory with section work, and even to be trained by a member of the honorary staff to give anæsthetics in cases of emergency, which they have done with marked success. Some of them also have given much appreciated assistance in the operation theatre. It is pleasant to hear that in the result the authorities have decided to pay each sister a war bonus so as to bring her salary up to £50 as a minimum.

One of our correspondents, a matron of a county hospital, regards this paper, we are glad to find, as full of interest and of much help to herself. She is anxious that the subject of the Laundry Department and its efficient management should be dealt with fully. Perhaps one or more of our readers who has made a special study of the laundry may be moved to send us an account of their work and experience in this branch of hospital management. Some Northern and the better Scottish hospitals place the laundry in charge of a sister who is technically qualified to work it thoroughly. We should welcome a communication from one of these ladies, whose efficiency we have had good reason to appreciate in the course of our inspections of several of the larger hospitals where their work does them honour. We mention these facts because this section can be made, by inter-communication and co-operation amongst hospital workers, one of the most practical and interesting sections of The Hospital.

Many of the voluntary hospitals which have set aside beds for the reception of wounded sailors or soldiers have at the moment an opportunity to carry through a little spring cleaning, owing to the temporary closing of some of these wards as the pressure of patients has diminished. In one institution in Scotland, where the number of wounded patients under treatment has filled eighty beds, the demand at the moment has so decreased that there are less than half that number at present under treatment. The removal of the former great pressure on the staff has given time for useful reflection, with the result that the directors are converting the attics of the new wing of one of the nurses' homes into a large play-room for the nurses. We are glad to hear that the chairman of the directors has shown his keen interest in the importance of this provision by furnishing the play-room throughout in a most suitable and attractive way. The nurses of this hospital are fortunate, for they will not only have a piano, but many varieties of table games, such as indoor croquet, cricket, ping-pong, and so forth. This thoughtful kindness is calculated to increase the efficiency and health of every member of the nursing staff.

Many matrons have experienced anxiety either from a shortage in the number of applicants for training as probationers, or from the fact that the standard of qualification in such candidates has not been as high as formerly. Experience since the war has proved, however, that these two problems are really interdependent. In some of the larger provincial hospitals the number of applicants for training shows an enormous increase, a fact which has proved helpful by enabling the matron to weed out with a drastic hand, and to choose the educated women to train as nurses. In one hospital the average monthly number of applicants for training since the war began has increased from thirty-five to 150. The result has been a refusal to admit resident pupils for short periods of training owing to an absence of accommodation; to raise the whole type of the probationers in training, with the ultimate, and possibly the immediate, result of greater efficiency in the working of the wards.

We are very appreciative of the generous help we are receiving from an increasing number of matrons and sisters to whom we are greatly indebted for the co-operation they have extended to this department of The Hospital. We are fully aware that the staff at military, territorial, and civil hospitals receiving wounded warriors are often over-pressed and over-worked. We have no desire to add to their burden in busy times, but those matrons and sisters who have any leaning to literary work might find a weekly note sent to The Hospital a help for its relaxation value, and the power it has to take their thoughts from the daily pressure under which they are too often obliged to live. Some matrons assure us that when they are busy again, and new developments and interests are thus created, they will be pleased to tell us of them. This is indeed good and welcome news.

